# ‘Reduced malignancy as a mechanism for longevity in mice with adenylyl cyclase type 5 disruption’

**DOI:** 10.1111/acel.12152

**Published:** 2013-10-13

**Authors:** Mariana S De Lorenzo, Wen Chen, Erdene Baljinnyam, María J Carlini, Krista La Perle, Sanford P Bishop, Thomas E Wagner, Arnold B Rabson, Dorothy E Vatner, Lydia I Puricelli, Stephen F Vatner

**Affiliations:** 1 Department of Cell Biology & Molecular Medicine and the Cardiovascular Research Institute, New Jersey Medical School, Rutgers University, The State University of New Jersey185 South Orange Avenue, MSB G609, Newark, NJ, 07103, USA; 2 Clemson UniversityClemson, SC, 29634, USA; 3 Instituto de Oncología ‘Ángel H. Roffo’Av. San Martín 5481, C1417DTB, Buenos Aires, Argentina; 4 Department of Veterinary Biosciences, College of Veterinary Medicine, The Ohio State University470 Veterinary Medicine Academic Building, 1900 Coffey Road, Columbus, OH, 43210, USA; 5 RWJMS, Rutgers, The State University of New Jersey89 French Street, 4th Floor, New Brunswick, NJ, 08901, USA

**Keywords:** AC5 inhibitor, adenylyl cyclase (AC) knockout mice, angiogenesis, B16F10 melanoma, LP07 lung adenocarcinoma, MMTV-HER-2 neu mice, tumor protection

## Abstract

Disruption of adenylyl cyclase type 5 (AC5) knockout (KO) is a novel model for longevity. Because malignancy is a major cause of death and reduced lifespan in mice, the goal of this investigation was to examine the role of AC5KO in protecting against cancer. There have been numerous discoveries in genetically engineered mice over the past several decades, but few have been translated to the bedside. One major reason is that it is difficult to alter a gene in patients, but rather a pharmacological approach is more appropriate. The current investigation employs a parallel construction to examine the extent to which inhibiting AC5, either in a genetic knockout (KO) or by a specific pharmacological inhibitor protects against cancer. This study is unique, not only because a combined genetic and pharmacological approach is rare, but also there are no prior studies on the extent to which AC5 affects cancer. We found that AC5KO delayed age-related tumor incidence significantly, as well as protecting against mammary tumor development in AC5KO × MMTV-HER-2 neu mice, and B16F10 melanoma tumor growth, which can explain why AC5KO is a model of longevity. In addition, a Food and Drug Administration approved antiviral agent, adenine 9-β-D-arabinofuranoside (Vidarabine or AraAde), which specifically inhibits AC5, reduces LP07 lung and B16F10 melanoma tumor growth in syngeneic mice. Thus, inhibition of AC5 is a previously unreported mechanism for prevention of cancers associated with aging and that can be targeted by an available pharmacologic inhibitor, with potential consequent extension of lifespan.

## Introduction

A significant impediment to improving cancer therapy is the relatively small numbers of new tumor-inhibitory mechanisms for which effective therapies can be developed for translation to patients. One potential source for discovering novel molecular mechanisms that protect against malignancy is a longevity model, because a major cause of premature death in mice can be attributed to this disease process. One such model, which we discovered and described, is the knockout of adenylyl cyclase type 5 (AC5). These mice live a third longer than wild-type (WT) littermates and are protected from the cardiomyopathy of aging (Yan *et al*., 2007). Because mice do not generally die of heart disease, but do die of malignancy, the first goal of this investigation was to determine whether knockout of AC5 (AC5KO) would protect against spontaneous tumors over the mouse lifespan and to determine its role in protection against induced tumors. This investigation identifies for the first time that knockout of this AC isoform protects against cancer. The results could not have been predicted, as no prior study has examined the role of AC5 in mediating tumor growth.

Adenylyl cyclase (AC) is a ubiquitous enzyme that catalyzes the synthesis of cAMP, activating PKA and the cAMP response element-binding protein (CREB) (Patel *et al*., 2001; Bos, 2003; Willoughby & Cooper, 2007). However, the results from previous studies have been controversial, some supporting and others discounting a role for this pathway in cancer (Schmidt *et al*., 1999; Subbaramaiah *et al*., 2006; Yano *et al*., 2007; Sakamoto & Frank, 2009; Chandramouli *et al*., 2010; Merkle & Hoffmann, 2011). AC is also a central component of the β-adrenergic-signaling pathway and interestingly a recent study identifies β-adrenergic receptor blockers as potential antitumor agents (Ji *et al*., 2012; Armaiz-Pena *et al*., 2013).

Our hypothesis is that the controversy with regard to AC effects on cancer may potentially be reconciled by identifying which of the nine major AC isoforms are involved. Each of these regulates function in different organs, and some of these may actually have opposite effects in regulation of disease processes. Over the past 25 years, there has been an explosion of novel transgenic and knockout models engineered in mice that have an adverse or salutary effect on the malignant disease process in the mouse, but relatively few have been translated to clinical application. There are several reasons why so few of these mechanisms have been translated to the bedside. First, there may be major species differences. A second, and potentially more important reason, is that it is difficult to translate disrupting a gene in mouse to human cancer therapy. To this end, it would be valuable to have a pharmacological analogue of the disrupted gene. We recently found that a drug, which was Food and Drug Administration (FDA) approved for antiviral therapy in patients, Vidarabine, also is a specific inhibitor of AC5 (Plunkett *et al*., 1974; Iwatsubo *et al*., 2012). Accordingly, the next major goal of this investigation was to determine whether this drug also protects against malignancy.

Several experimental approaches were employed to prove our hypothesis: first was to examine retrospectively the incidence of tumors over the life of AC5KO mice and WT littermates; the second was prospective, that is, to determine whether mating the AC5KO mice with MMTV-HER-2 neu mice, which are known to be prone to develop mammary tumors (Jacquemart *et al*., 2009), would induce protection in the bigenic mice; the third was to examine to which extent AC5KO mice were protected against syngeneic melanoma tumor growth; the final goal was to conduct a parallel investigation on the extent to which the antiviral drug, Vidarabine, which selectively inhibits AC5 (Plunkett *et al*., 1974; Iwatsubo *et al*., 2012), also protects against malignancy. Our studies demonstrate that inhibition of AC5 may represent a novel approach to inhibiting spontaneous cancers associated with aging and mortality.

## Results

**Figure 1:**
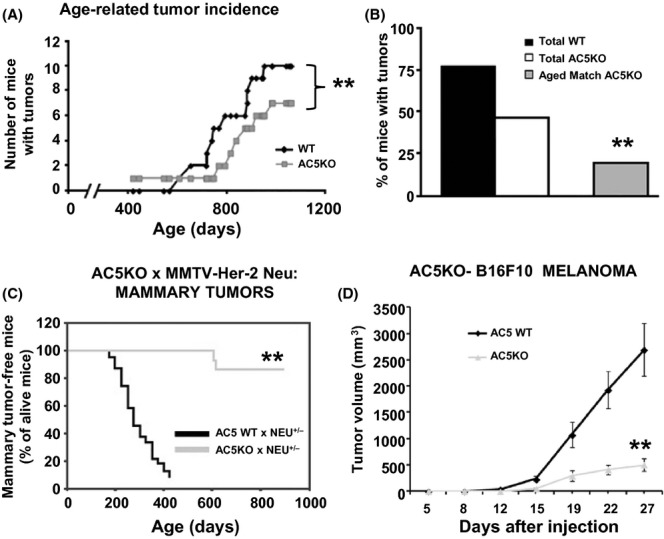
AC5KO mice have a delayed tumor onset. (A) A retrospective study using female and male mice demonstrated a significantly reduced age-related tumor incidence in AC5KO mice, **P < 0.01 by log-rank test and by chi-square test; *n* = 13–15. This figure presents the tumor incidence in the mice undergoing autopsy. The *y*-axis represents the accumulated tumor incidence and the *x*-axis represents the age of mice when tumor was observed. This graph demonstrated that the tumor incidence was delayed in AC5KO mice. (B) The data from Fig. A were replotted comparing the percentage of animals demonstrating tumors at autopsy for wild-type (WT), all the AC5KO mice and the smaller subgroup of AC5KO mice that died of similar lifespan as WT (bar at the right). The percentage of AC5KO mice with tumors found at autopsy was significantly less (*P* < 0.01) than in WT. (C) Genetically modified AC5KO × Neu^+/−^ mice also have delayed age-associated tumor development and reduced mammary tumor incidence, **P < 0.01 by log-rank test and by chi-square test; *n* = 13–15, compared with WT × Neu^+/−^ mice (See also Fig. S1). (D) AC5KO mice also showed protection against B16F10 melanoma tumor growth. Tumor growth was significantly reduced in AC5KO mice; **P < 0.01 vs. AC5WT.

Our retrospective analysis demonstrated that AC5KO exhibited a significant delay in age-related tumor incidence (*P* < 0.01; Fig. 1A), which can help explain the prolonged longevity in this model (Yan *et al*., 2007). The most common type of tumors found were lymphomas, histiocytic sarcomas, bronchial alveolar carcinomas, schwannomas, and hepatocarcinomas. Figure 1B shows the percentage of mice that died with tumors. Clearly, the numbers were dramatically less in AC5KO and even more so when only mice of the same age at the time of death were compared (Fig. 1B bar at the right).

The second goal was prospective, that is, to determine whether mating the AC5KO mice with MMTV-HER-2 neu mice, which are known to spontaneously develop mammary tumors (Jacquemart *et al*., 2009), would induce protection in the bigenic mice. We found that AC5KO × MMTV-Her-2 neu showed a significant delayed age-related tumor development (*P* < 0.01) and a lower mammary tumor incidence (Fig. 1C) compared with AC5WT × MMTV-Her-2 neu (*P* < 0.01). The results were striking: by day 521 all WT MMTV-Her-2 neu mice developed mammary carcinomas and died, but there were no deaths in AC5KO-MMTV-Her-2 neu at that time (Fig. 1C). Representative histological examples of tumors are shown (Fig. S1, Supporting information).

It is well known that obesity and diabetes are established risk factors for the development of postmenopausal breast cancer and are associated with the increased risk of other cancers, for example, endometrium, kidney, colon, and esophagus (Calle *et al*., 2003; Shikata *et al*., 2013). Because AC5KO mice have lower body weight and adiposity index than WT and have better glucose tolerance and insulin resistance (Yan *et al*., 2007, 2012; Vatner *et al*., 2013); these factors may mediate AC5KO cancer protection. AC5KO mice also have lower leptin levels (AC5KO: 1.99 ± 0.28 vs. WT: 3.23 ± 0.35 ng mL^−1^; *P* < 0.05) and we confirmed that AC5KO × MMTV-Her-2 neu mice have significantly lower body weight (*P* < 0.01).

The third goal was to examine the extent to which AC5KO mice are protected against syngeneic tumor growth for which we used the standard B16F10 melanoma model. After B16F10 melanoma cells of C57BL/6 origin were inoculated s.c., AC5KO demonstrated decreased tumor growth compared with AC5WT (*P* < 0.05 vs. AC5WT). These results were striking: at day 27, tumor volume in WT was 6-fold greater than in AC5KO (Fig. 1D). Tumor latency was doubled in AC5KO vs. WT (14.1 vs. 6.6 days, *P* < 0.01), while average wet tumor weight was less than half in AC5KO (*P* < 0.01). These data suggest that the effects of AC5KO might be not only at the level of cellular transformation, but also at the level of tumor growth and progression.

**Figure 2:**
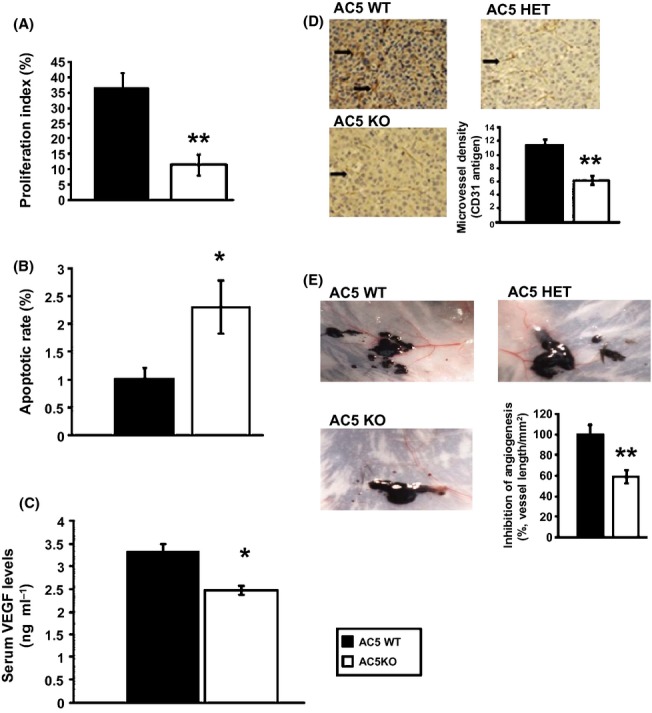
Genetic disruption of adenylyl cyclase type 5 (AC5) protects against melanoma. Tumors from AC5KO mice demonstrated significant protection against melanoma. AC5KO showed decreased cell proliferation index compared with AC5WT mice (A), increased apoptosis in melanoma tumors (by TUNEL assay) (B), and reduced serum VEGF levels (C). Angiogenesis is reduced in B16F10 melanoma in AC5KO mice, as reflected by reduced microvascular density, with examples shown in photomicrographs and quantitation in the bar graph (D), and reduced vessel length around tumor implants, and with representative photographs showing B16F10-induced angiogenesis in each group of mice (E). **P < 0.01 and *P < 0.05 vs. AC5WT; *n* = 4.

We next assessed the mechanisms responsible for reduced B16F10 melanoma growth. AC5KO mice were protected through several mechanisms that regulate tumor growth, for example, angiogenesis, apoptosis, and tumor cell proliferation (Hanahan & Weinberg, 2000; Hanahan & Weinberg, 2011). The cell proliferation index was reduced by over half, and the apoptotic rate was more than doubled in AC5KO tumors compared with AC5WT (Fig. 2A,B). AC5KO mice are also protected from tumor angiogenesis and had significantly lower levels of circulating VEGF in serum compared with AC5WT (Fig. 2C) and tumor microvascular density (Fig. 2D); *P* < 0.01. Tumors from AC5KO mice exhibited less angiogenesis *in vivo*, as measured by total vessel length (Fig. 2E, *bottom right*), *P* < 0.01.

**Figure 3:**
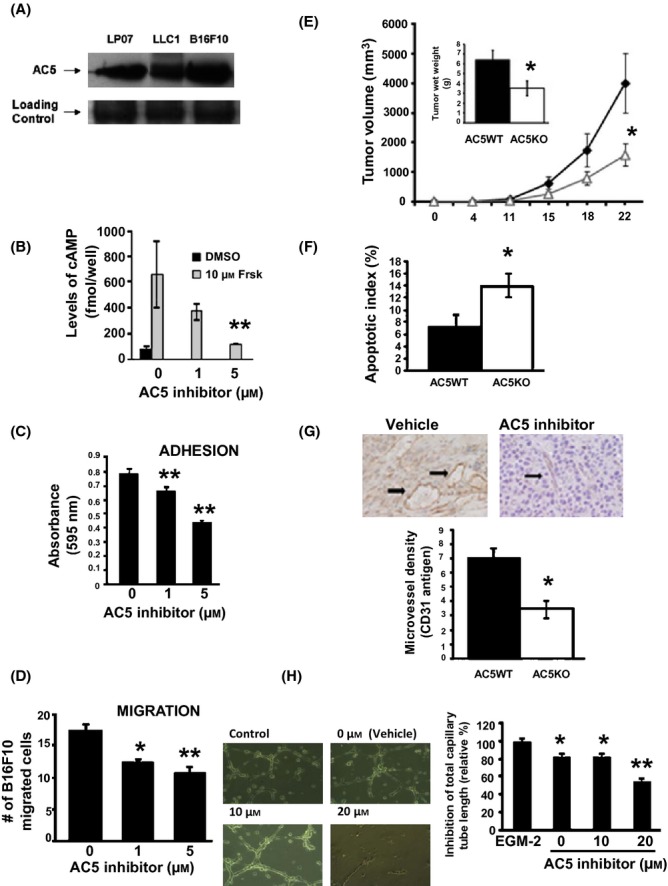
Pharmacological inhibition of adenylyl cyclase type 5 (AC5) reduces B16F10 mouse melanoma growth. (A) AC5 protein is expressed in LP07 and LLC1 lung carcinoma and B16F10 melanoma cells. (B) B16F10 tumor cells were pretreated for 10 min in basal medium with the AC5 inhibitor (0–5 μm) and then treated with forskolin or vehicle for 15 min. Forskolin (FrsK, 10 μm) increase in cAMP was reduced by AC5 inhibitor in a dose-dependent manner in B16F10 cells. The AC5 inhibitor reduced *in vitro* tumor cell adhesion (C) and migration (D). (E) Treatment with the AC5 inhibitor, 50 mg kg^−1^ day^−1^, reduced melanoma tumor growth after 8 × 10^4^ B16F10 melanoma cells were inoculated subcutaneously in the flank of C57B16 mice. Tumor wet weights were significantly different. *P < 0.05 vs. vehicle; *n* = 12 per group *(*E*, small inset)*. (F) The AC5 inhibitor increased the intratumor apoptotic index; *P < 0.05 vs. vehicle; *n* = 5. (G) The AC5 inhibitor reduced B16F10 melanoma angiogenesis as reflected by reduced microvascular density (arrows indicate microvessels in the tumor) and reduced endothelial capillary tube formation, with representative examples shown in (H) (left) and quantitative data in (H) (right). *P < 0.05 and **P < 0.01 vs. EGM-2 medium and vehicle; *n* = 3–12.

**Figure 4:**
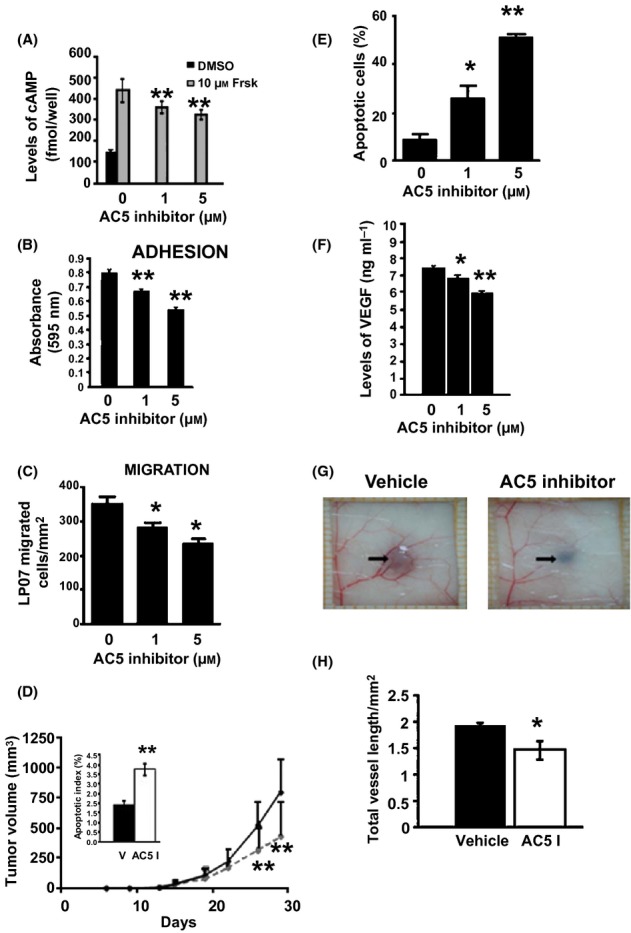
Treatment with adenylyl cyclase type 5 (AC5) inhibitor (50 mg kg^−1^ day^−1^) reduces lung tumor growth. LP07 cells were pretreated for 10 min in basal medium with AC5 inhibitor (0–5 μm), which reduced forskolin-induced cAMP production (A); adhesive capacity of LPO7 cells (B); migratory capacity of LPO7 cells (C), apoptosis (E); and basal secretion of VEGF (F). (D) Tumor growth induced by LP07 cells was reduced by the AC5 inhibitor, dotted line, which also increased the intratumor apoptotic index (inset). (G) Representative pictures from the vascular areas surrounding growing tumors induced by injected LPO7 cells (black arrows indicate the tumor implantation site) show that the AC5 inhibitor reduced intratumor microvessel density (G), with quantitation in (H). **P < 0.01 and *P < 0.05 vs. vehicle; *n* = 3–9.

A fourth goal was to conduct a parallel investigation on the extent to which the antiviral drug, Vidarabine, which selectively inhibits AC5 (Plunkett *et al*., 1974; Iwatsubo *et al*., 2012) also protects against malignancy. Treatment of B16F10 (Fig. 3B) and LP07 cells (Fig. 4A) with the AC5 inhibitor reduced forskolin-induced cAMP production (*P* < 0.01), demonstrating its ability to block endogenous AC in these tumor cells. Inhibiting cAMP reduces expression of PKA-CREB-dependent genes involved in angiogenesis, inflammation, and tumor growth (Sakamoto & Frank, 2009; Hanahan & Weinberg, 2011) and treatment with the AC5 inhibitor produced dose-dependent reductions in B16F10, LP07, and Lewis lung carcinoma cells (LLC1) cell adhesion and migratory capacity (Fig. 3C,D; Fig. 4B,C; Fig. S2E,F, Supporting information), that is, additional mechanisms meditating tumor growth and progression. Our results show that pharmacological AC5 inhibition protects against tumor growth and against both tumor cell and host mechanisms facilitating tumor growth (proliferation, adhesion, migration, angiogenesis) in syngeneic mouse models and in murine tumor cell lines; for example, LP07 lung adenocarcinoma (Urtreger *et al*., 2001; Peluffo *et al*., 2004), LLC1, B16F10 melanoma cells (Fig. S2). Using a specific type 5 AC monoclonal antibody, we showed the expression of AC5 in B16F10, LP07, and LLC1 cells (Fig. 3A), AC5 inhibitor reduced *in vitro* cell proliferation (Fig. S2A,B,C).

In the syngeneic mouse models, the AC5 inhibitor reduced B16F10 (Fig. 3E) and LP07 (Fig. 4D) tumor growth rate and tumor weight (Fig. 3E *inset*) compared with vehicle-treated mice (*P* < 0.05 and *P* < 0.05). We measured the intratumor cell proliferation by nuclear staining of Ki-67 antigen and observed that tumors from AC5 inhibitor-treated mice exhibited a significantly reduced cell proliferative index (*P* < 0.01). In addition, the AC5 inhibitor doubled the apoptotic index in B16F10 tumors (Fig. 3F) and in LP07 tumors (Fig. 4D, *inset*). We also found that the percentage of cells with apoptosis was increased in LP07 cells treated with the AC5 inhibitor (Fig. 4E) along with other indices of apoptosis, the higher percentage of cells in pre-G_0_ (Fig. S3A, Supporting information); the expression of Annexin V (Fig. S3B), and the increased marker of apoptosis, cleaved caspase-3, along with a concomitant decrease in the expression of Bcl-2, a known antiapoptotic marker (Fig. S3C).

The AC5 inhibitor also protected against tumor angiogenesis. Staining of tumors with CD31 after 22 days of treatment showed that intratumor microvessel density was reduced by 50% (Fig. 3G, *lower graph*). To elucidate the effects of the AC5 inhibitor on endothelial cells, we used HUVEC cells that have a population doubling time of approximately 24 h and found that the viability of HUVEC was reduced with the AC5 inhibitor (50 μm) after 24 h (*P* < 0.01 vs. vehicle; Fig. S2D). The AC5 inhibitor also caused a dose-dependent inhibition of endothelial cell capillary tube formation, which was performed on Matrigel (Fig. 3H, *right side*).

One mechanism mediating the reduced angiogenesis with the AC5 inhibitor was VEGF. We also found that AC5 inhibitor treatment reduced the basal secretion of VEGF (Fig. 4F; *P* < 0.05) and the forskolin-induced secretion (*P* < 0.01). Angiogenesis was also evaluated by staining the tumors after 30 days of treatment with the AC5 inhibitor or vehicle with CD31 and found reduced microvascular density. Lastly, *in vivo* angiogenesis was also evaluated. The density and length of vessels induced by the LP07 tumors were reduced in AC5 inhibitor-treated mice (*P* < 0.05; Fig. 4H).

## Discussion

There are two major findings in the current investigation. The first is that disruption of AC5 protects against tumor growth, and the second is that similar results are observed with a pharmacological inhibitor of AC5. We identified for the first time that inhibition of a specific isoform of AC, that is, AC5 protected against cancer. This is significant for several reasons and may help resolve the prior controversy in the literature, as prior studies did not examine specific isoforms and as different isoforms may have different or even opposite effects on any given disease process. The second major finding is that a pharmacological AC5 inhibitor, Vidarabine, replicates the salutary effects of AC5 disruption. This is significant, as this allows translation to patients with cancer either with this compound or one that is engineered for safety and efficacy by the pharmaceutical industry.

The current investigation was stimulated by our previous study, which demonstrated increased longevity in AC5KO mice (Yan *et al*., 2007). Because a major cause of death in mice is neoplastic disease, we reasoned syllogistically that AC5KO mice may be protected from this cause of death, which in turn, protected against premature mortality. Accordingly, our first goal was to determine the time of death in these mice and their WT littermates and that were related to tumors. We found that AC5KO mice have a delayed age-related tumor incidence, and the most common neoplasms were lymphomas, pulmonary adenomas, histiocytic sarcomas, hepatocarcinomas, schwannomas, and bronchial alveolar adenomas.

Our next goal was to determine whether the AC5KO mice were protected from induced tumors. To address this question, we studied MMTV-Her-2 neu mice, which develop mammary tumors (Jacquemart *et al*., 2009). We found that disruption of AC5 leads to a significant protection against age-related tumors and to a reduction in the incidence of mammary tumors in AC5KO × MMTV-Her-2 neu mice, compared with controls, which was consistent with our findings of protection against all naturally developing tumors in aging AC5KO and WT mice. Our AC5KO × MMTV-Her-2 neu mice displayed a reduced incidence of mammary tumors, but also lymphomas, histiocytic sarcomas, and hepatocellular adenomas and carcinomas.

Another feature of AC5KO mice is their protection from obesity, diabetes, and oxidative stress factors associated with increased cancer incidence (Yan *et al*., 2007, 2012; Vatner *et al*., 2013). This is important for interpretation of the current results, as it is well known that obesity is an established risk factor for the development of postmenopausal breast cancer and is associated with the increased risk for other cancers in endometrium, kidney, colon, and esophagus. Diabetes has also been associated with increased incidence of cancer (Shikata *et al*., 2013). Importantly, we previously reported that AC5KO mice have lower body weight and adiposity indices than WT (Yan *et al*., 2012) and lower levels of the hormone leptin. Whereas diabetes and obesity are major causes of morbidity and reduced longevity in humans (Knight, 2011), in mice cancer is a more important cause of mortality (Storer, 1966; Wolf *et al*., 1988; Chrisp *et al*., 1996). The data from the current investigation support the concept that AC5 inhibition reduces cancer and by syllogistic reasoning may be responsible in part for the extended longevity in this model. The third model employed involved grafting of B16F10 melanoma cells to the mice. AC5KO mice showed reduced tumor growth compared with WT in this model as well. The reduction in tumor growth was accompanied by significantly reduced angiogenesis and increased tumor cell apoptosis in AC5KO mice, two mechanisms that protect against tumor growth (Hanahan & Weinberg, 2011).

Finally, and potentially most interesting, we found that the antiviral drug, Vidarabine, previously used in this country and still used in others for Herpes treatment in patients (De Clercq, 1993), has selective AC5 inhibitory properties (Iwatsubo *et al*., 2012), and that this drug mimics the salutary effects of AC5 disruption on protection against cancer. The drug protected against tumor growth in syngeneic mouse models and in murine tumor cell lines; for example, LP07 lung adenocarcinoma, LLC1 and B16F10 melanoma cells. It also enhanced mechanisms known to protect against tumor growth, that is, by reducing proliferation, adhesion, migration, and angiogenesis, while increasing apoptosis. As noted above the protection induced by the AC5KO and by the AC5 inhibitor, both involved angiogenic mechanisms, a key regulator of tumor growth. The data on VEGF and reduced tumor vascular density support this concept. Although this drug inhibits AC5 strongly, but total AC activity weakly, suggesting a specific AC5 inhibitor, it is possible that there is inhibition of other AC isoforms, but unlikely as potent as inhibition of AC5. One limitation of the studies with the AC5 inhibitor is that experiments were not extended to old mice. It remains to be determined if this pharmacological approach mimics the AC5KO model and extends longevity and reduces cancer incidence in old mice.

In summary, this investigation demonstrates a novel mechanism for cancer protection, inhibition of AC5, which is unique not only because this has not been described previously, but also because parallel studies with a pharmacological inhibitor also supports a protective role for inhibiting AC5. Because the pharmacological inhibitor is already FDA approved, the journey from bench to bedside should not be long. The effects of AC5 inhibition in protecting against obesity, diabetes, and oxidative stress as well as enhancing exercise capacity, all further support its potential utility in cancer treatment and/or prevention, as diabetes and obesity and sedentary lifestyle have all been linked to cancer. Because cancer is a major cause of death in mice (Storer, 1966; Wolf *et al*., 1988; Chrisp *et al*., 1996) and as we demonstrated less cancer in the AC5KO mice, then by syllogistic reasoning, these findings may provide an explanation for the longevity in the AC5KO model. Whereas this is the first report of a specific AC isoform to exert an effect on cancer, the prior studies on AC (not isoform specific) have reported controversial results. This may be because different AC isoforms exert different effects on cancer. In the future, it will be important to investigate this hypothesis with different specific AC isoform KO models.

## Experimental procedures

### Cell culture and reagents

LP07 lung adenocarcinoma cell line was obtained *in vitro* from a s.c. passage of the original spontaneous P07 tumor and cultured in MEM supplemented with 10% FBS, 2 mm L-glutamine and 80 μg mL^−1^ gentamycin (Urtreger *et al*., 2001; Peluffo *et al*., 2004). B16F10 melanoma and Lewis lung carcinoma LLC1 cells (ATCC, Manassas, VA, USA) and human umbilical vein endothelial cells (HUVEC; Lonza Walkersville Inc., Walkersville, MD, USA) were cultured as described by the manufactures. Adenine 9-β-D-arabinofuranoside (AraAde, Vidarabine or AC5 inhibitor) was purchased from Sigma (St. Louis, MO, USA) and was dissolved in DMSO as vehicle (0 μm) for *in vitro* experiments.

### Mice

AC5KO mice on C57BL/6 or mixed 129SVJ/C57BL/6 background were used. FVB/N Tg (MMTVneu) 202Mul/J mice, expressing the WT rat neu transgene under the control of the mouse mammary tumor promoter (MMTV-neu), were obtained from Jackson Laboratories (Bar Harbor, ME). AC5 (WT, HET, KO) mixed 129SVJ/C57BL/6 background, and AC5 (WT, HET, KO) × MMTV-neu female mice were obtained and monitored for tumor incidence along their life. BALB/c and C57BL6, 2–4 month-old mice were used for the short-term mouse tumor experiments. They were weight-matched and kept in our Animal Care Division. Mice were housed in a pathogen-free facility under a 12:12 h light–dark cycle with access to water and food *ad libitum*. Studies were approved by the Animal Care and Use Committees of Clemson University, Instituto de Oncologia ‘Angel H. Roffo’ and New Jersey Medical School.

### Tumor experiments

(A) AC5 (WT, HET, KO) experiments: 8 × 10^4^ B16F10 cells (C57BL/6 origin) suspended in 0.2 mL medium were injected s.c into the right flank of mice (*n* = 4–9/group). (B) AC5 inhibitor on B16F10 melanoma growth: miniosmotic pumps (ALZET model 2004, DURECT Corporation) containing vehicle or AC5 inhibitor (50 mg kg^−1^ day^−1^) were implanted s.c in C57BL6 mice 3 days after being treated; 8 × 10^4^ B16F10 were injected into the right flank of mice (*n* = 14/group) and treatments continued till the end of the experiment. (C) AC5 inhibitor on LP07 lung cancer growth: LP07 cells (3 × 10^5^) were inoculated s.c. in the right flank of mice BALB/c (*n* = 9). When tumors became palpable, animals were treated daily with Vidarabine (50 mg kg^−1^ day^−1^) or vehicle intraperitoneally during 30 days. Vehicle was a 0.25% sodium carboxymethylcellulose solution that was stored at −20 °C, thawed overnight, and completed with povidone stock solution (4.25%, kept at 4 °C) to a 0.085% final concentration. Vidarabine (50 mg kg^−1^ day^−1^) or vehicle was prepared weekly, and a magnetic stir bar was added to the bottle to keep the suspension phase throughout the dosing. Tumor growth was assessed by measurement of tumor diameter in the longest dimension (L) and at right angles to that axis (Width, W) using a Vernier caliper. Tumor volumes were estimated using the formula, L × W × W × π/6.

### Pathology

Half of each tumor was fixed in 10% phosphate-buffered formalin, dehydrated, and embedded in paraffin. Major organs were subjected to gross pathology, and tumor slides were stained with haematoxylin-eosin. Mammary tumors were graded in accordance with criteria of Cardiff *et al*. (2000). For the analysis of lymphomas, the evaluation criteria were described by Kogan *et al*. (2002).

### *In vivo* angiogenesis

Ten-week-old female AC5WT, AC5HET, AC5KO mice were injected intradermally in the dorsal midscapular part of mice (*n* = 5–12/group) with 8 × 10^4^ B16F10 cells in 250 μL DMEM plus trypan blue (De Lorenzo *et al*., 2003) and LP07 (1 × 10^5^) cells in BALB/c mice (*n* = 5/group) in 0.1 mL MEM plus trypan blue to localize the site of inoculation. After 7 days, mice receiving Vidarabine (50 mg kg^−1^ day^−1^) or vehicle were euthanized and the inner skin was exposed, and the vascular response was observed with a dissecting microscope using 6.5× magnification. Inoculated sites were photographed using a high-resolution camera. A microscope micrometer was used to calibrate length in mm. The total length of vessels was calculated based on the calibration by the Image Pro Plus software. *In vivo* angiogenesis was represented by the total length of vessels around the tumors and expressed as percentage of inhibition compared with AC5WT or vehicle-treated mice.

### Proliferation and microvessel density

Tumor cell proliferation and angiogenesis were evaluated by immunostaining for Ki-67 (Neo Markers, Fremont, CA, USA) and CD31 (Santa Cruz Biotechnology Inc., Santa Cruz, CA, USA) proteins, respectively. Proliferation was assessed by counting the number of tumor cells with positive nuclei (for Ki-67) at 400× magnification. Ten fields were counted per sample and results were expressed as the proliferation index: proportion of the positively staining cells over the total number of cells. Microvessel density was assessed by counting the number of CD31 positive vessels at 400× magnification in the fields with the highest vascularization (De Lorenzo *et al*., 2011).

### TUNEL assay

Apoptosis was measured by using terminal deoxyribonucleotide transferase (TdT)-mediated dUTP nick-end labeling (TUNEL) (Roche Diagnostics, Indianapolis, IN, USA) as described previously (De Lorenzo *et al*., 2011).

### Immunoassays

VEGF and leptin protein levels were measured by Quantikine immunoassay kits (R&D Systems, Minneapolis, MN, USA) following manufacturer’s recommendations.

### Western blot analysis

The expression of AC5 in tumor cell lines was quantified using a monoclonal antibody as described previously (Hu *et al*., 2009). For the detection of the apoptotic markers, tumor cell monolayers were lysed and sonicated in RIPA lysis buffer containing 25 mm Tris–HCl (pH 7.6), 150 mm NaCl, 1% NP-40, 1% sodium deoxycholate, 0.1% SDS with phosphatase inhibitor cocktails 1 and 2 (Sigma), protease inhibitor cocktail (Thermo Scientific), and 1 mm NaF. Equal amounts of protein (20–90 μg) were subjected to SDS-PAGE. After electrophoresis, samples were transferred to millipore immobilon-P membrane and immunoblotted with antibodies against β-actin (as an internal loading control), Bcl-2, total caspase-3 (Santa Cruz Biotechnologies), and cleaved caspase-3 (Cell Signaling).

### Cell proliferation assay

LP07, B16F10, LLC1 (ATCC), and HUVEC were seeded in 96-well (5 × 10^3^), flat-bottomed plates in 10% FBS MEM or DMEM media or 2% FBS EGM-2 and allowed to adhere overnight. Then, fresh medium containing vehicle or Vidarabine (0–50 μm) in serum-free or 2% FBS medium was added. At 24–72 h, the culture medium was replaced with 3-[4, 5-dimethylthiazol-2-yl]-2.5-diphenyltetrazolium bromide (MTT, 0.5 mg mL^−1^). The reaction was stopped 4 h later by removing the medium and then adding 100 μL DMSO. Absorbance was measured after 30 min at 560 nm in a 96-well plate reader. IC_50_ values were calculated using GraphPad Prism software (GraphPad Software, La Jolla, CA, USA).

### cAMP accumulation assay

cAMP levels in LP07 and B16F10 cells were measured by Biotrak™ enzyme-immunoassay (GE Healthcare). 2.5 × 10^3^ LP07 lung or 2 × 10^3^ B16F10 melanoma cells were seeded in 96-well plates and incubated overnight. Different doses of Vidarabine or vehicle were used and manufacturer’s recommendations were followed to measure cAMP concentration. Absorbance was measured using a spectrophotometer at 450 nm.

### Cell adhesion

10^5^ LP07, LLC1, or B16F10 cells were seeded in 96-well plates in basal medium containing vehicle or AC5 inhibitor (0–5 μm) and incubated for 90 min. Then, the medium was discarded; cells were fixed, and stained with 100 μL of methanol/crystal violet solution. After 3 h of incubation, wells were washed with PBS and then cells were lysed with 250 μL SDS. Absorbance was measured with spectrophotometer at 595 nm.

### Cell migration

(A) LP07 subconfluent monolayers were pretreated overnight with 2% FBS MEM containing vehicle or Vidarabine (0–5 μm) and 10^5^ LLC1 or B16F10 cells were seeded on top of the membrane of each transwell insert in basal DMEM with vehicle or AC5 inhibitor (0–5 μm), and migration was evaluated as described previously (Urtreger *et al*., 2001; De Lorenzo *et al*., 2004).

### Endothelial capillary tube formation

HUVEC capillary network *in vitro* was evaluated as previously described (De Lorenzo *et al*., 2004). Briefly, Matrigel™ was placed onto 24-well plates and allowed to gel. HUVEC were grown on 2% FBS- EGM-2 medium. 5 × 10^4^ cells were added per well and cultured in the presence of vehicle or AC5 inhibitor (0–20 μm) overnight. Using the Imagine Pro Plus software, the total capillary tube length was calculated.

### Cell cycle analysis

LP07 cells were treated with vehicle or Vidarabine (0–5 μm) for 24 and 48 h. Then, cells were trypsinized, washed, suspended in ice-cold PBS (pH 7.4), counted, and fixed overnight in 50% ethanol at 4 °C. Then, (10^6^ mL^−1^) cells were resuspended in 1.12% sodium citrate with RNase A (500 units mL^−1^) for 30 min at 37 °C and then propidium iodide (50 μg mL^−1^) was added. Red fluorescence of single events was recorded using an argon ion laser at 488-nm excitation wavelength and 610-nm emission wavelength to measure DNA index on a Coulter Epics XL flow cytometer. The percentage of cells present in each phase of the cell cycle was determined using ModFitLT V2.0 software from Verity Software House (Topsham, ME, USA). The percentage of apoptotic cells as well as cells in G0/G1, S, and G2/M phases was calculated.

### Evaluation of apoptotic cells by nuclear staining

LP07 cells were treated with vehicle or Vidarabine (0–5 μm). Thirty-six hours later, cover slips were stained with DAPI and mounted on slides with PBS: Glycerol (1:1). Cells were observed for nuclear condensation/fragmentation as an indicator of apoptosis with a fluorescent microscope. Results were expressed as the percentage of total apoptotic cells vs. total number of cells.

### Annexin-V FITC

Apoptosis was also quantified by flow cytometry by AnnexinV/PI staining using the Annexin V: fluorescein isothiocyanate Apoptosis Detection Kit I (BD-Pharmingen, San Diego CA, USA).

### Statistical analysis

Statistical comparisons were performed by spss (IBM Corporation, Armonk, NY, USA) and GraphPad InStat (GraphPad Software Inc., La Jolla, CA, USA) softwares. Log-rank, Chi-square test, Student’s *t-*test, and Fisher exact test were used, and one-way analysis of variance (ANOVA) was used when comparing more than two groups. After ANOVA, Tukey–Kramer multiple comparisons test was used. The level of statistical significance for all tests was *P* < 0.05.

## Supporting Information

**Fig. S1** Genetically modified AC5KO crossed with MMTV-Her-2-Neu mice. **Fig. S2** AC5 inhibitor reduces cell proliferation, adhesion and migration. **Fig. S3** AC5 inhibitor increases apoptosis in LP07 cells.
