# Supplementary material for: ‘Reduced malignancy as a mechanism for longevity in mice with adenylyl cyclase type 5 disruption’
Source: Aging Cell. 2013 Oct 13;13(1):102–10. doi: 10.1111/acel.12152 (PMC3980454; doi:10.1111/acel.12152)
Supplement: Supplementary file 1 — Fig. S1 Genetically modified AC5KO crossed with MMTV-Her-2-Neu mice. Fig. S2 AC5 inhibitor reduces cell proliferation, adhesion and migration. Fig. S3 AC5 inhibitor increases apoptosis in LP07 cells. [file acel0013-0102-sd1.ppt]

## Slide 1
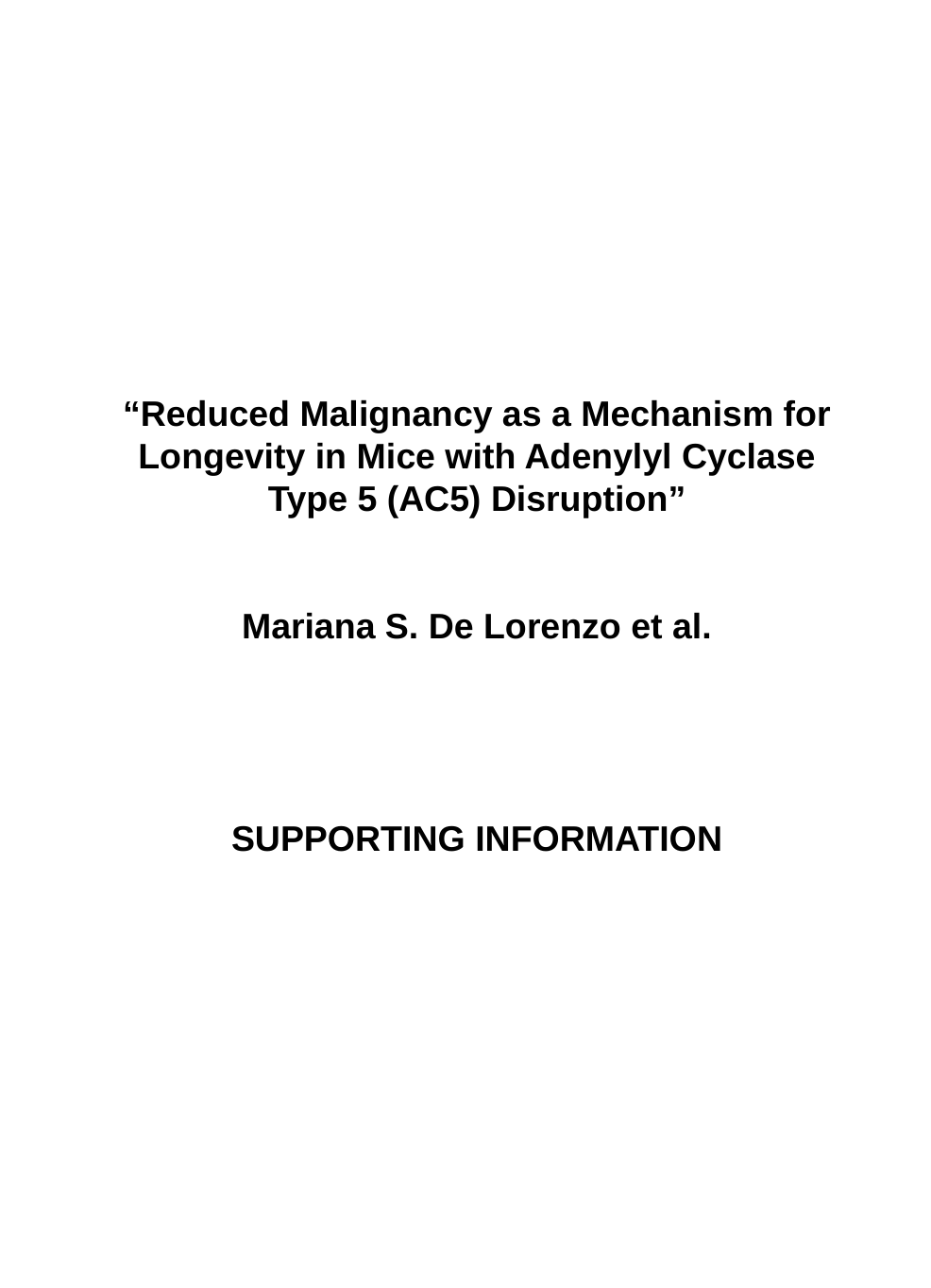

“Reduced Malignancy as a Mechanism for Longevity in Mice with Adenylyl Cyclase Type 5 (AC5) Disruption”
Mariana S. De Lorenzo et al.
SUPPORTING INFORMATION

## Slide 2
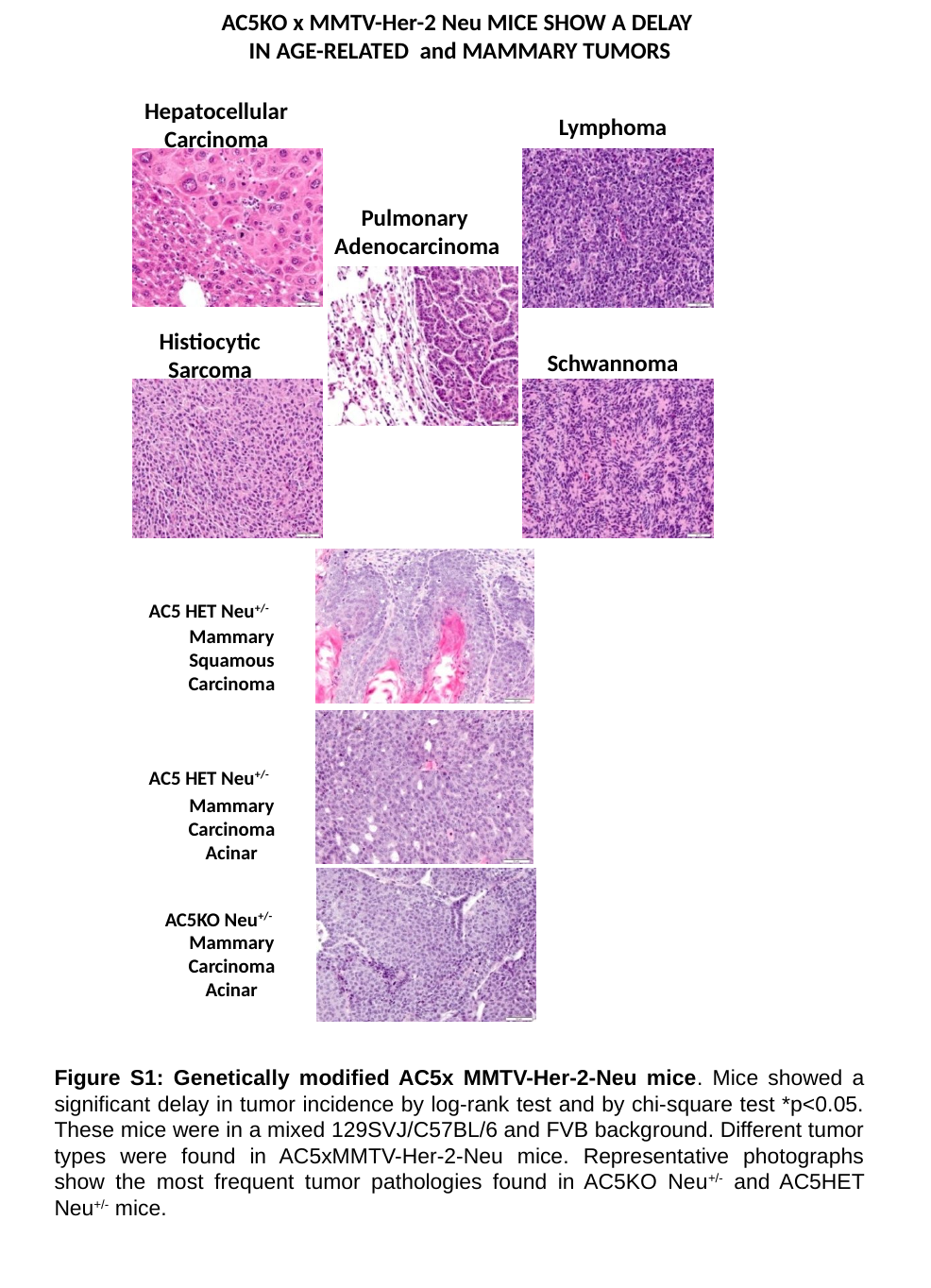

AC5KO x MMTV-Her-2 Neu MICE SHOW A DELAY
 IN AGE-RELATED and MAMMARY TUMORS
Hepatocellular
Carcinoma
Lymphoma
Pulmonary
Adenocarcinoma
Histiocytic
Sarcoma
 Schwannoma
AC5 HET Neu+/-
Mammary
Squamous
Carcinoma
AC5 HET Neu+/-
Mammary
Carcinoma
Acinar
AC5KO Neu+/-
Mammary
Carcinoma
Acinar
Figure S1: Genetically modified AC5x MMTV-Her-2-Neu mice. Mice showed a significant delay in tumor incidence by log-rank test and by chi-square test *p<0.05. These mice were in a mixed 129SVJ/C57BL/6 and FVB background. Different tumor types were found in AC5xMMTV-Her-2-Neu mice. Representative photographs show the most frequent tumor pathologies found in AC5KO Neu+/- and AC5HET Neu+/- mice.

## Slide 3
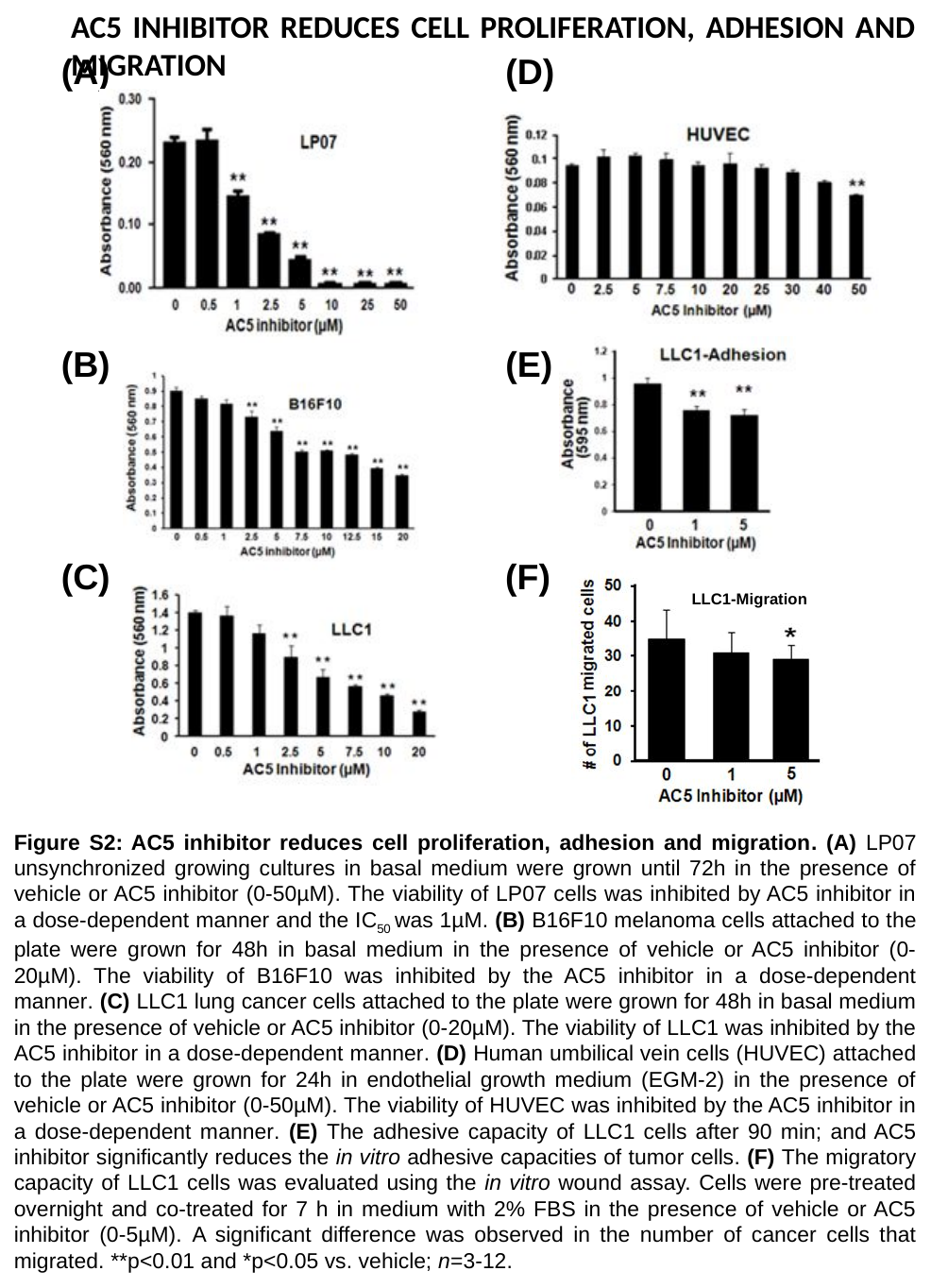

AC5 INHIBITOR REDUCES CELL PROLIFERATION, ADHESION AND MIGRATION
(A)
(D)
(B)
(E)
(C)
(F)
LLC1-Migration
Figure S2: AC5 inhibitor reduces cell proliferation, adhesion and migration. (A) LP07 unsynchronized growing cultures in basal medium were grown until 72h in the presence of vehicle or AC5 inhibitor (0-50µM). The viability of LP07 cells was inhibited by AC5 inhibitor in a dose-dependent manner and the IC50 was 1µM. (B) B16F10 melanoma cells attached to the plate were grown for 48h in basal medium in the presence of vehicle or AC5 inhibitor (0-20µM). The viability of B16F10 was inhibited by the AC5 inhibitor in a dose-dependent manner. (C) LLC1 lung cancer cells attached to the plate were grown for 48h in basal medium in the presence of vehicle or AC5 inhibitor (0-20µM). The viability of LLC1 was inhibited by the AC5 inhibitor in a dose-dependent manner. (D) Human umbilical vein cells (HUVEC) attached to the plate were grown for 24h in endothelial growth medium (EGM-2) in the presence of vehicle or AC5 inhibitor (0-50µM). The viability of HUVEC was inhibited by the AC5 inhibitor in a dose-dependent manner. (E) The adhesive capacity of LLC1 cells after 90 min; and AC5 inhibitor significantly reduces the in vitro adhesive capacities of tumor cells. (F) The migratory capacity of LLC1 cells was evaluated using the in vitro wound assay. Cells were pre-treated overnight and co-treated for 7 h in medium with 2% FBS in the presence of vehicle or AC5 inhibitor (0-5µM). A significant difference was observed in the number of cancer cells that migrated. **p<0.01 and *p<0.05 vs. vehicle; n=3-12.

## Slide 4
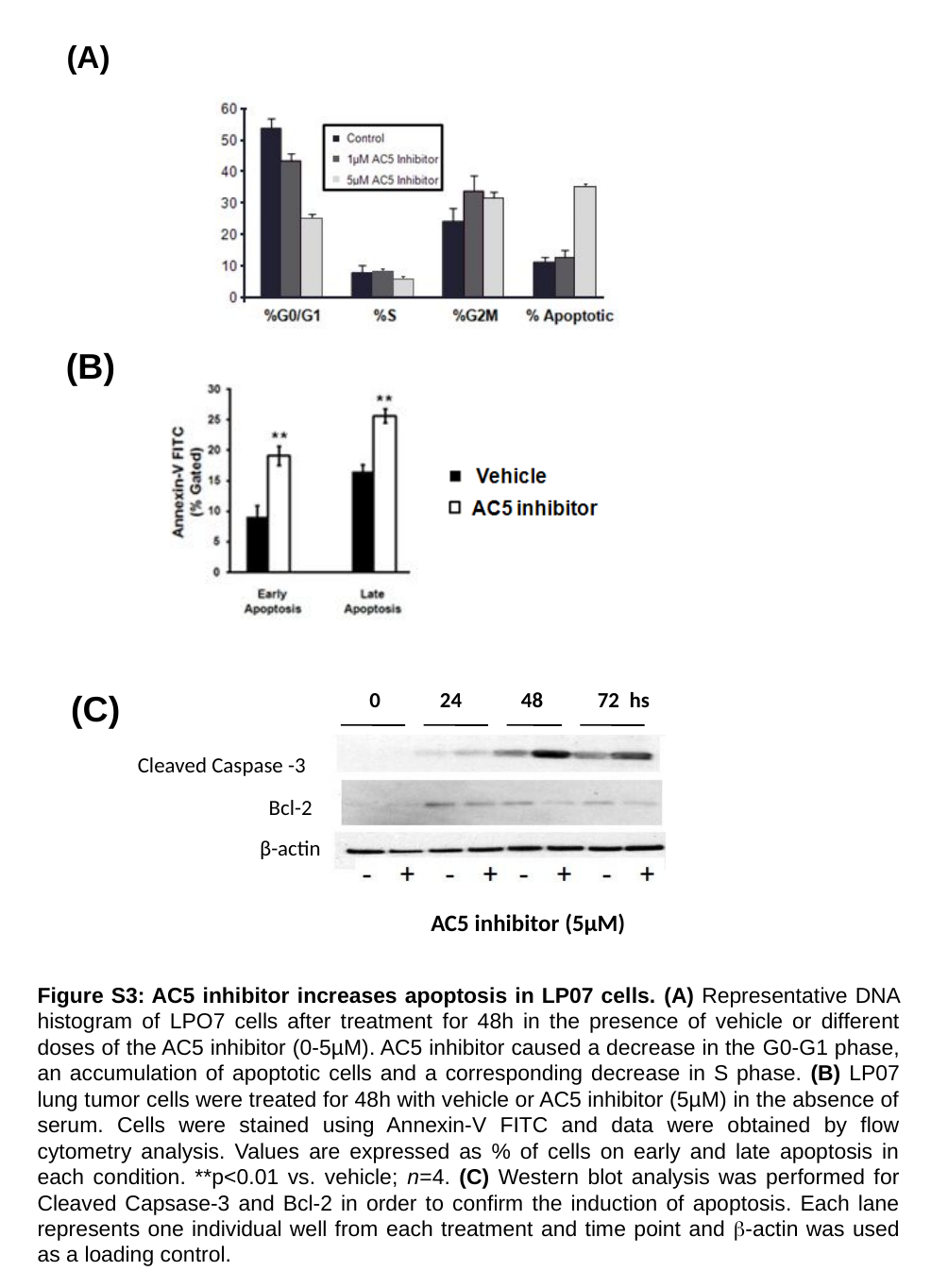

(A)
(B)
(C)
 0 24 48 72 hs
Cleaved Caspase -3
Bcl-2
β-actin
AC5 inhibitor (5µM)
Figure S3: AC5 inhibitor increases apoptosis in LP07 cells. (A) Representative DNA histogram of LPO7 cells after treatment for 48h in the presence of vehicle or different doses of the AC5 inhibitor (0-5µM). AC5 inhibitor caused a decrease in the G0-G1 phase, an accumulation of apoptotic cells and a corresponding decrease in S phase. (B) LP07 lung tumor cells were treated for 48h with vehicle or AC5 inhibitor (5µM) in the absence of serum. Cells were stained using Annexin-V FITC and data were obtained by flow cytometry analysis. Values are expressed as % of cells on early and late apoptosis in each condition. **p<0.01 vs. vehicle; n=4. (C) Western blot analysis was performed for Cleaved Capsase-3 and Bcl-2 in order to confirm the induction of apoptosis. Each lane represents one individual well from each treatment and time point and β-actin was used as a loading control.
